# Quality of care as perceived by caregivers and residents in aged care homes in Sri Lanka: a qualitative study

**DOI:** 10.1186/s12877-024-04721-y

**Published:** 2024-01-27

**Authors:** Madushika Wishvanie Kodagoda Gamage, Hemamali Madhushanthi Hirimbura Jagodage, KKPM Kumari De Silva, Pramudika Nirmani Kariyawasam, Thamudi Dharshi Sundarapperuma

**Affiliations:** Department of Nursing, Faculty of Allied Health Sciences, University of Ruhuna, Matara, Sri Lanka

**Keywords:** Quality of care, Older people, Aged care homes, Sri Lanka, Qualitative study

## Abstract

**Objective:**

Quality of care determines the physical and psychological wellbeing of aged care residents. The study aims to explore barriers and facilitators of quality of care in aged care homes (ACHs) in Sri Lanka from the perspective of older adults and caregivers.

**Methods:**

This qualitative descriptive exploratory study was conducted in selected five ACHs in Galle District, Sri Lanka, from May 2021 to January 2022. We recruited a purposive sample of residents and caregivers and conducted ten in-depth interviews with ten caregivers and nine focus group discussions with forty-five residents. The data were analysed using the thematic analysis.

**Results:**

The majority of caregivers and residents were females. Caregivers and residents were aged 25-35 years and 70-80 years, respectively. Two major themes emerged: (a) barriers of quality of care and (b) facilitators of quality of care. Both residents and caregivers reported a lack of referring system facilities; a lack of financial support on the infrastructure; a lack of financial support in supplying medication and employing human resources; insufficient knowledge of gerontological care and geriatric syndromes for both caregivers and residents; and lack of interest in being a caregiver at the ACHs as barriers in providing quality care. Moreover, caregivers and older people reported donations by philanthropists; supportive leadership; and welfare benefits from the government authorities as facilitators of quality care. Additionally, caregivers reported a lack of in-service programmes for caregivers as a barrier and positive institutional values as a facilitator for providing quality care.

**Conclusion:**

The available human and physical resources are insufficient to address the demanding needs of residents in ACHs, apart from the received donations. There is a simultaneous need for healthcare policymakers' and social welfare authorities' attention to implementing necessary measures to uplift the quality of care for residents in ACHs to enhance their quality of life.

## Introduction

Population ageing is a growing concern due to global health, economic, and social burdens. The older population (i.e., above 60 years) is approximately 770 million worldwide. The older population will probably exceed two billion by 2050 [1]. Sri Lanka, one of the fastest ageing countries, is experiencing an overwhelming proportion of older people over 60 years compared to other South Asian countries [2]. The proportion of the older population in Sri Lanka is predicted to double from 16% in 2020 to 30% in 2050 [3].

Population ageing is a significant burden in Sri Lanka, and suboptimal resources are available to address the needs of an ageing society [4]. Moreover, it has various economic, health, and social repercussions and implications. Providing sustained care for older people is difficult in Sri Lankan society, along with alterations in family structure and work patterns [5]. This results in increased admissions to aged care homes (ACHs). As a result, the number of ACHs and day-care centres in Sri Lanka has increased over the last two decades. About 300 ACHs [6] and 147 day-care centres are within the country [7].

The quality of care in ACHs is a priority concern with the increased number of admissions. The quality of care, a broader concept, describes the 'goal that health and aged care services should seek to provide'. The extent to which the health services meet the people's desires as they expect is often described as the quality of care. According to the World Health Organisation, high-quality care involves safe, person-centred, effective, timely, efficient, and equitable care [8]. The individuals' physical, psychosocial, and spiritual needs should be accomplished, and their health outcomes need to be improved in the sense that they receive quality care service. Quality of care is an important determinant of actualising better health and improving the quality of life and the satisfaction of older residents [9].

Various factors influence the quality of care in ACHs, and poor quality of care will lead to negative consequences. Empirical evidence suggests a range of barriers to quality of care, including minor mistakes, discontinuity, multiple care providers [8], frequent policy and regulatory changes, heavy workloads of the staff, high staff turnover, difficult recruitment, and challenges in retaining competent staff [9]. Diminished quality of care in ACHs could lead to consequences, such as adverse safety events. For example, accidental injuries involving residents and staff, pressure ulcers, falls, wounds, and medication errors are the most common adverse safety events in ACHs [10, 11]. These consequences highlight the importance of enhancing the quality of care in ACHs by overcoming barriers. Caregivers play an important role in providing quality care for residents in the ACHs. Mainly, caregivers assess medical needs, assist with basic needs, and provide companionship and emotional support [12] as the regular carers of residents. Though the quality of care in a care home is an important parameter, only a limited number of quality improvement initiatives have been developed and tested in the care homes [9].

There is limited understanding of residents' quality of care in ACHs in the literature. Studies assessing this aspect are limited, and it has yet to be discovered in Sri Lanka. Procedures to assess quality of care do not exist yet in Sri Lanka, irrespective of increased admissions to ACHs. Therefore, exploring perceptions of quality of care through a qualitative study will add value to the available literature. As caregivers provide care for residents in ACHs, the perception of both groups will provide a broader understanding. The study findings will facilitate the identification of barriers and, therefore, can be used to develop future mechanisms to improve the quality of care. Hence, this study explores different aspects related to the quality of care for residents and caregivers.

## Aim

The study explores barriers and facilitators of quality of care in aged care homes in Sri Lanka from the perspective of older adults and caregivers.

## Methods

### Study design and settings

This study was a community-based, qualitative, descriptive exploratory study carried out in some selected ACHs in Sri Lanka. According to the census, the older adult population, and the number of ACHs are highest in Western and Southern provinces [4], so Galle District, a district in the Southern province, was selected for the study. There are 21 registered ACHs in Galle District, and five ACHs were selected randomly. To select five homes randomly, 21 tickets mentioning the name of each ACH were tossed. A person outside the research team randomly selected the names of five homes.

### Study participants

Caregivers and residents in ACHs who can speak and understand English or Sinhala (narrative language) were purposefully recruited for this study. Further, priority was given to caregivers with more than one year of working experience and residents with no psychological health problems. All eligible caregivers and residents were verbally invited, and written informed consent was obtained before recruiting them to the study. There were around two to three caregivers in an ACH. Therefore, we recruited all caregivers in five ACHs who fulfilled the above criteria. When recruiting residents, we obtained a list of older people who fulfilled the above criteria (i.e., without any psychological health problems and willing to participate in the study). From the list, we purposefully selected older people to receive a maximum variation for demographic characteristics, such as age, gender, and presence of diseases.

### Data collection

In-depth interviews (IDIs) were conducted to assess caregivers' perspectives because IDIs allow detailed exploration of respondents' reactions without contamination [13]. Further, IDIs ensured longer speaking time and privacy during information gathering. IDIs were conducted until new information was no longer generated. Ten caregivers were recruited from ACHs. Ten IDIs were conducted; that is, one for each caregiver, each lasting approximately 60-90 minutes.

Focus group discussions (FGDs) were used to explore residents' perceptions about the quality of care to allow cross-talk between them and generate ideas freely. Forty-five volunteering residents in ACHs were recruited. Three to five residents were invited for each FGD, as smaller groups are more manageable to obtain optimum results [14]. Nine FGDs were carried out until new information was no longer generated; data saturation was achieved [15], with each session lasting for 30–60 minutes [13].

Two separate semi-structured guides developed by the research team facilitated FGDs and IDIs. The guides were developed using the literature findings, the expert advice of a psychologist, and a geriatric physician. The discussion guides were rephrased and finalised after pilot testing them with five residents and two caregivers. The discussions and interviews were conducted in quiet, comfortable rooms in ACHs. The research team members facilitated all sessions after having an in-depth discussion about the role of the moderator. The notetaker recorded all nonverbal and verbal expressions [16]. All sessions were audio-recorded and transcribed verbatim [16].

### Data analysis

The thematic analysis method was used for data analysis [17], and we used an inductive approach. The entire research team checked all transcripts for errors by reading them and listening to the audio recordings simultaneously. After familiarising with transcripts, the coding was started. The data of the nineteen transcripts (ten of IDIs and nine of FGDs) were coded separately to minimise potential bias. The process of refining and applying was repeated until no new codes were generated [17]. The themes were generated once all data had been coded [17]. The generated themes were reviewed to recheck the appropriateness of the themes [17]. Themes were reported after defining and naming themes [17].

### Rigor

To ensure of trustworthiness of data, the criteria of credibility, confirmability, dependability, and transferability were established [18]. All the interviews were transcribed verbatim by the research team to help with immersion in the data. All transcripts were translated into English and discussed with the research team. Collectively, these measures assisted with establishing the credibility of this study [18]. During data analysis, a codebook was prepared and the decisions made during analysis were recorded in a notebook; these strategies assisted with establishing confirmability [18]. Dependability was demonstrated by the researcher documenting detailed information about the study setting, participants, data collection process, and analytical steps. Finally, transferability was enabled through the detailed descriptions of participants' experiences in the findings [18].

### Ethical consideration

Ethical clearance was obtained from the Ethics review committee, Faculty of Allied Health Sciences, University of Ruhuna, Sri Lanka (Ref No: 27.02.2020:3.5). Participants were invited to participate and informed written consent was obtained. Participants' rights were respected. Anonymity and confidentiality were maintained using an identification serial number for each participant. The research team only had access to the participant's information. There was no conflict of interest among the investigators, nor was there any social, financial, or legal issue. Voluntary participation was highly expected. The participants were allowed to withdraw from the research study without giving reasons. Data were collected without interfering with the routine activities of the ACHs.

## Results

### Participant characteristics

The ten caregivers who participated in the IDIs were in the age category range of 25 to 35 years and had a diploma or above. Additionally, the majority were females (80%). The nine focus groups involved 45 residents aged between 70 to 80 years. Most residents were women (75.5%), had non-communicable disease (95%), and had no income (66.7%). Almost all the participants were Sinhalese Buddhists. The participant characteristics are as shown in Table 1.

**Table 1:** Participant characteristics

**Characteristics**	**Caregiver (*N*=10)**	**Residents (*N*=45)**
Gender		
Female	08	34
Male	02	11
Age		
25–35 years	10	
35–55 years		
55–65 years		
More than 65 years		45
Education level		
Below grade 5		30
Up to Ordinary level		10
Up to Advanced level		4
Diploma/Degree/or above	10	1
Mode of income		
Salary	10	
Pension		3
Insurance		2
From family members		3
Government allowance		7
No income		30
Duration in the ACH		
1–5 years	7	10
5–10 years	2	13
More than 10 years	1	22

**Fig. 1:**
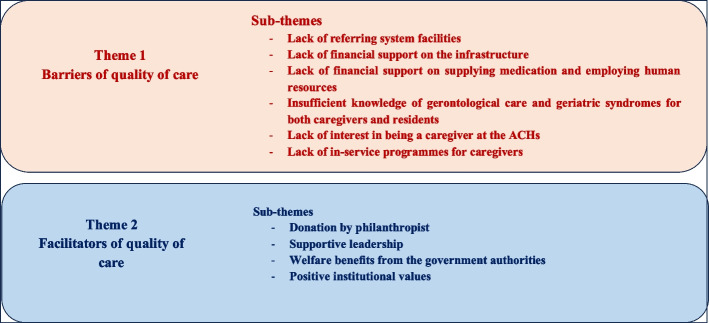
Themes and subthemes

Two major themes emerged from focus group discussions and in-depth interviews: (a) barriers of quality of care (i.e., the factors that suppress quality care) and (b) facilitators of quality of care (i.e., the factors that enhance the quality of care of residents). We found five sub-themes under the theme barriers of quality care. Among them, five subthemes emerged both from older people and caregivers, namely lack of referring system facilities; lack of financial support on the infrastructure; lack of financial support on supplying medication and employing human resources; insufficient knowledge of gerontological care and geriatric syndromes for both caregivers and residents; and lack of interest in being a caregiver at the ACHs. Meanwhile, one subtheme, the lack of in-service programmes for caregivers, emerged only from the discussions with caregivers. Additionally, we identified four subthemes under the theme facilitators of quality care emerged from caregivers and older people: donations by philanthropists, supportive leadership, and welfare benefits from the government authorities. One subtheme, positive institutional values, surfaced solely from FGDs with caregivers (see Fig. 1).

## Theme one: barriers of quality of care

### Lack of referring system facilities

Conventionally, almost all the caregivers described the insufficiency of facilities, more importantly, insufficient referring system facilities, as a barrier. Since a considerable proportion of participants live with chronic illnesses, accessible medical care was a key variable in ascertaining the quality of care as perceived by caregivers working in ACHs. However, there was no appointed medical personal to refer residents and had no adequate transportation facilities to transfer residents to hospital or clinics, when needed, impeding the quality of care.

*An appointed medical person is not available in our home. There is no easy access to health care, although almost all residents seek medical attention (CG5).*

*The Karapitiya Hospital is nearest to our aged care home, but our residents do not have transportation facilities to participate in routine clinics (CG2).*

*Most of the time, I hire a three-wheel and pay by myself to transport them to the hospital and medical centres (CG6).*

All the aged care residents who participated in the study endorsed the statements made by the caregivers regarding the available facilities. Residents frequently discussed about not receiving a tangible support from caregivers.

*We cannot request to appoint a medical officer or request vehicles to go to our routine clinics. Sometimes, Ayurvedic doctors come to our homes, but we are unsatisfied with them. However, we are now worthless people (R2, focus group 1).*

*Sometimes, we go long distances to measure our blood pressure and dress our wounds since we do not have a trained healthcare person in our home (R3, focus group 5).*

### Lack of financial support on the infrastructure

Caregivers discussed financial issues as a barrier for good infrastructure. Almost all caregivers emphasised the availability of insufficient funds to sustain adequate facilities and to fulfil the additional needs of residents during the in-depth interviews.

*These buildings are very old, so it is important to renovate them; on some rainy days, water flows inside, but it is difficult to find money these days because all have financial problems (CG1).*

Residents reaffirmed the caregivers' views on the lack of financial support on the infrastructure.

*We wrote several letters to authorised people requesting help to renovate these buildings. They informed us that the government had not allocated money for such activities and advised us to utilise our funds or donations, but, unfortunately, we are running this home with many financial difficulties. We have to spend all our funds on food and medications for our older adults as few donations get these days due to the COVID outbreak (CG9).*

### Lack of financial support on supplying medication and employing human resources

Caregivers explained that funds for residents are inadequate even to afford essential needs, such as drugs and some nutrient supplements. Also, caregivers explained that they do not have adequate human resources to care for older people.

*We receive a small sum of money from the social service department for residents. It is at least not enough to buy their medicines. Sometimes, we need money to buy essential things for residents, such as medicines, plasters, supplements, etc., but we do not have adequate money to buy them (CG8).*

*Though hiring a good helper to clean washrooms is important, it is not affordable; therefore, sometimes, we ask our residents to clean washrooms and own rooms (CG7).*

Residents reaffirmed the caregivers’ views on the perceived lack of support in supplying medication and employing human resources.

*Now, we are not earning, and we do not have children or supporters to look after us. So, we depend on caregivers even to buy very small things and also important things, such as pills. Some of our fellow residents get small sums from the social service department, but this sum is inadequate to buy things (R31, focus group 7).*

*We are cleaning washrooms as well as our living areas. Sometimes, we are the helpers for our sick friends. Though we have several body aches and difficulties, we must do these things because the aged care home does not have enough money to pay additional workers (R37, focus group 8).*

### Insufficient knowledge of gerontological care and geriatric syndromes for both caregivers and residents

Caregivers' knowledge about the ageing process, including physical and psychological changes, is important for quality care provision and for promoting the health of older residents. However, caregivers reported inadequate knowledge of age-related diseases, including behavioural changes and memory loss.

*I have heard that older adults’ behaviour can change drastically along with their ageing process. Activities, including puzzle-solving, slow down behavioural changes, and memory loss. Truly, I do not have sound knowledge of that. I have two or three residents with total memory loss, but I do not know how to provide care for them. Sometimes I feel exhausted (CG3).*

Further, caregivers verbalised their inadequate knowledge and uncertainty about common physical diseases related to ageing.

*I have heard that diabetes is a disease of older people and giving them special food to control their sugar limit is good. I do not know how I should change their diet plan (CG4).*

Similarly, residents reported that they do not have an in-depth understanding of the ageing process, including changes in cognition.

*I have heard that memory is going to fade with age. However, I do not know what actions are necessary to attenuate the progression of this process (R12, focus group 3).*

Further, residents reiterated that caregivers do not have adequate knowledge regarding diseases as to their observations, saying,

*Although our Mrs (caregiver) is unaware, she performs some treatments when we have physical illnesses, but she does not know much about memory loss (R19, focus group 4).*

*They know nothing about how they should handle a person with memory loss. They do not know enough about suitable food for some disease conditions such as diabetes, hypertension, etc. So, they supply common food for all (R8, focus group 2).*

### Lack of interest in being a caregiver at the ACHs

Willingness is the major potentiality of the person to be a caregiver. Caregivers affirmed that most people do not like to be a caregiver, and caregiver burnout is high, specifically with lower wages and rest periods. One caregiver depicted,

*We get a small allowance, which is inadequate to look after our families. So, most caregivers are unwilling to be a caregiver (CG1).*

With the absence of leave and inadequate flexibility in working hours, most Sri Lankan caregivers miss their family commitments with their job roles. With insufficient human resources, replacement of duties is rarely possible. Caregivers stated,

*This is not an eight-hour duty. We must work 24 hours. Sometimes, we need to spend sleepless nights. Sometimes, we cannot find time for family commitments. Though we need to see our family members, sometimes there is nobody to hand over our duties (CG2).*

Residents verbalised the impact of frequent changes of caregiving person on their day-to-day life.

*We love some caregivers, but we are sad when they leave soon. When they change, we must introduce ourselves again and again to the new people. Sometimes, it makes me embarrassed (R27, focus group 6).*

### Lack of in-service programmes for caregivers

Another concern expressed by caregivers was the lack of in-service programmes to uplift their knowledge and skills. They were willing to learn more about the caregiving process and basic skills.

*I only know how to look after them by my experience. I do not have any special training or basic training in caregiving, and sometimes, I do not have much knowledge to care for them (CG10).*

It was observed that caregivers have identified their knowledge gap and have a factual interest in learning.

*I do not know any activity for mental health promotion. I do not know how to handle residents with memory loss, but I am really interested in learning about them (CG5).*

## Theme two: Facilitators of quality of care

### Donation by philanthropist

Caregivers indicated that religious belief in donations is one of the major facilitators of quality care. The majority of the Sri Lankans are Buddhists. In Buddhist culture, giving something to a needy person is treated as a merit for both this birth and the next birth. Caregivers explained that Sri Lankans' belief in "Karma" (i.e., merit for the next life) is the main reason for donations, such as food and clothes.

*The majority of donors believe that donation is a precious thing to enhance their next life and prosperity of this life. Hence, they try to donate food and clothes as much as possible to celebrate important life events (CG7).*

*Before this COVID pandemic, we had at least one or two daily almsgivings (meal donations). Some of the donors provide new clothes with meals (CG8).*

The residents also agreed with the statements and verbalised that they receive good meals when somebody gives alms. Residents are satisfied with receiving at least some food for all the meals. Sometimes, they were unhappy about the meal's content as they were willing to eat different food.

*We get delicious food for some days, and some donors give us clothes, reading materials, and herbal medicines (R33, focus group 7).*

*If there is no almsgiving, our in-charge Mrs. (caregiver) cook for us with our support. So, every time we have something to eat. Though we do not have any choice to select food, we are happy because we have enough food to survive (R8, focus group 2).*

### Supportive leadership

The caregivers explained that the administrative staff provides optimum support to take necessary actions to enhance the friendly atmosphere and quality of care.

*Our chair (Owner or manager) has permitted us to do any good activity for older adults’ health. We have changed the environment in a better way with the support of our residents. Our chair is impressed with it. The Board of committee members of this ACH gives their maximum help to enhance the quality of this institution. They have given us autonomy (CG9).*

Residents endorsed the statements made by caregivers. They indicated that the heads of these institutions are very compassionate regarding residents, so they always help the caregivers and residents to change the environment and other things as they wish. Therefore, residents said it gives them more relaxation and freedom.

*Yes, this is not our home or a familiar place, but we have to spend the last stage of our life journey here. So, freedom or mental relaxation is an immense need. We have it here because the owner of this ACH is very caring, so they provide their maximum support to our caregivers if they plan to perform good activities for us (R43, focus group 9).*

### Welfare benefits from the government authorities

Government authorities are responsible for looking after older people in ACHs. Officers of these authorities mainly monitor the functions of the ACHs regularly, but the government does not provide enough money to maintain the quality of care of the ACH. The government only provides a very small sum monthly for the residents’ diet, but it is not enough at least to provide one meal for residents. However, caregivers highly admire the support, continuous follow-ups, and coordinating activities of the government institutions, which help to enhance the quality of care for older people.

*These officers regularly visit our place, make us aware of the latest updates and help us find solutions to any prevailing problems (CG3).*

*Some counsellors and volunteers, especially foreigners, are introduced to our institution, and these volunteers try their best to enhance the mental and physical wellbeing of residents, but, frankly, we cannot satisfy the allocated money from the government to maintain ACH. They always ask us to find donors (CG4).*

Residents also confirmed the statements of caregivers.

*The government officers are very kind and helpful. They come to see us frequently and try to help as much as possible. Suppose they identify any matter in our homes. In that case, they try their best to solve it, and they are directly involved in getting support from the government to enhance the quality of our lives and the quality of the ACH (R16, focus group 4).*

### Positive institutional values

This sub-theme emerged mainly from caregivers. Caregivers explained institutional values as a major facilitator to commence new health promotion programmes. They verbalised their positive attitudes towards initiating these programmes for older people and their willingness to support these programmes even though they faced such financial constraints.

*Our sir (chairperson of this institution) is always willing to support any programme to enhance the care for older adults, though we have faced a severe financial crisis due to covid outbreak (CG10).*

*We are also really enthusiastic to start an exercise programme or cognitive enhancement programme. Currently, to our knowledge, we ask residents to engage in physical activities as much as possible. However, providing a healthy and balanced diet is difficult due to financial issues (CG6).*

We did not identify differences in residents' or older people's interpretations of barriers and facilitators based on their cognitive abilities or background factors.

## Discussion

The older population in the whole globe is increasing. For instance, Sri Lanka has one of the fastest ageing populations in Asia [6]. In the Sri Lankan context, more older people are moving to ACHs parallel to the increasing ageing population. Aged care homes operate under government funds and provide their services free of charge to their residents. Caregivers provide care for older people living in ACHs [19]. This study explored the perception of the quality of care among residents and caregivers in an Asian cultural setting. Two major themes emerged: (a) barriers of quality of care and (b) facilitators of quality of care. We found six sub-themes under the theme barriers of quality care, namely lack of referring system facilities, lack of financial support on the infrastructure, lack of financial support on supplying medication and employing human resources, insufficient knowledge of gerontological care and geriatric syndromes for both caregivers and residents, lack of interest in being a caregiver at the ACHs, and lack of in-service programmes for caregivers. Additionally, we identified four subthemes under the theme facilitators of quality care: donation by philanthropists, supportive leadership, welfare benefits from the government authorities, and positive institutional values.

With ageing, there is a decline in mental and physical capacities, consequently increasing the disease risk [2]. Additionally, ageing is linked with other life transitions, including retirement and relocation [2]. For instance, demographic changes within the society are one of the reasons for increased relocation to ACHs. Previously, women predominantly assumed the responsibility of being housewives and caring for older people, but now most are busy with their occupations. Additionally, a reduction in the number of offspring, and migration searching for economic prosperity, busy lives, and poverty reduces the care for older people [19].

A qualitative holistic approach was used to study the perception of quality of care among residents and caregivers. Both stakeholders are key in determining the quality of care, which led to the inclusion of both parties within one study. Using surveys to study care quality demonstrates weaknesses, including a tendency to frame into categories based on the researcher's preconceived ideas [20]. Therefore, we used a qualitative approach in this study. We found subthemes on barriers and facilitators of quality of care from the perspective of both caregivers and residents. Facilitators enhance the quality of care, while barriers lessen the quality. To our knowledge, studies incorporating the voices of residents and caregivers to understand the perception of quality of care in aged care homes are scarce [20].

A recent review explored aged care residents' perspectives on quality of care and identified staffing levels and staff attitudes as factors influencing quality of care [21]. The review reported that residents often verbalised the inadequate number of staff members and sympathised with the staff situation. In our study, the lack of financial support for employing human resources was a barrier of quality care. This may lead to an inadequate number of caregivers to provide quality care for older people. However, we found positive institutional values as a facilitator of quality of care. Therefore, these values may help to increase the quality of care despite inadequate human resources. Caregivers in our study were ready to adapt to changes for the better wellbeing of older people. This finding could be a cultural norm which is found in many Asian communities who offer support for older individuals to relive their worries and stressors. Furthermore, supportive leadership and continuous support from the government authorities might encourage the changes.

Previous literature reported high staff turnover in ACHs, influencing continuity of care [21]. Informants in one study reported job security of the staff and professional expertise as essential elements to the quality of care. Moreover, high staff turnover was reported as a barrier of quality care [20]. Similarly in our study, caregivers affirmed that most people dislike being a caregiver, and a high caregiver burnout. Our study mentioned lower wages and inadequate leave as reasons for high turnover. Additionally, residents verbalised how these changes influence their daily activities. Supporting, residents always prefer familiar staff as it allows them to share personal stories and information [21]. Thus, targeted strategies are required retain and avoid burnout in caregivers. These strategies could include recruiting adequate human resources to care for older people and programs to support psychological well-being of caregivers. However, already existing financial constraints may limit the employment of human resources. Therefore, the other measures could be strengthening human resources within the ACH. This could be by allowing older people to be more independent in their homes. Older people in our study verbalised that they help the daily functioning of the ACH and help the other residents who cannot perform activities of daily living. This would make older people more physically active and reduce caregivers' workload. Additionally, helping older people stay more physically active could enhance their wellbeing and quality of life [22].

Our study residents were concerned about caregivers' training and highlighted the caregivers' need for more knowledge of gerontological care and geriatric syndromes. However, residents did not highlight the importance of trained professional caregivers in one study [20]. Staff knowledge, skills, and attitudes; interventions to improve staff capacity; staff wellbeing and workforce stability; and environmental factors that affect staff capacity were found to influence the quality of care in ACHs [23]. In Sri Lanka, the caregivers are not trained personnel and have fewer training opportunities. The lack of in-service programmes for caregivers was verbalised in our study. ACHs have a role to take measures to enhance the capacity building of caregivers. This could be through collaboration with educational institutions, such as Sri Lankan universities, that could run programmes to enhance caregivers’ skills and knowledge.

Our study reported a lack of referring system facilities inhibiting their access to healthcare. Older populations generally have multimorbidities [24], and access to healthcare is pivotal for this population. In the Sri Lankan context, physical illnesses, such as hypertension, diabetes mellitus, arthritis, and asthma, are common among people aged over 60 years [25]. Therefore, it is important to take measures to enhance these facilities in ACHs. The older person’s health is a significant factor that determines the opportunities and contributions of older individuals [2]. Policymakers should consider this aspect to improve the wellbeing of older people in care homes by increasing access to healthcare. Additionally, future research is encouraged to identify the most common diseases prevailing among older people in ACHs and the associated factors for this high prevalence. Moreover, caregivers and older people verbalised a lack of financial support for supplying medication. A recent review reported that medication adherence is lowest in young and significantly older people. Socioeconomic status and social support positively impact on adherence [26]. Therefore, financial constraints could lead to poor medication adherence.

Residents reported the built environment as a quality-of-care indicator, and residents with private rooms enjoyed privacy and valued the ability to fill rooms with personal possessions [21]. In one study, residents reported that shared rooms increased conflicts and violence [27]. In contrast, our infrastructure is not well developed for older people in care homes, as discussed by both residents and caregivers. We found lack of financial support for the infrastructure as the reason for not being able to provide them a home-like environment. The importance of having a supportive environment for older people was highlighted by WHO [2] as it facilitates opportunities despite losses in capacity.

Residents valued the freedom to decide about their care and treatments. For instance, residents valued the ability to express food preferences and inputs into food options [21]. Our study reported financial difficulties, which may limit the ability to make preferences for older people living in ACHs in Sri Lanka. However, donations from philanthropist (e.g., food) might have given opportunities to enjoy their preferences.

It was prominent that financial constraints had emerged as several barriers to quality of care. Therefore, finding solutions to make ACHs more financially independent institutions is important. Both older people and caregivers verbalised donations by philanthropists and welfare benefits from the government authorities as facilitators of quality of care. However, ACHs ought to take measures to enhance the capacity building of caregivers and residents to generate income. This will enhance the effective utilisation of free time, instead of waiting for donations and government support. Additionally, these activities could promote satisfaction and engagement in ACHs [21]. A review reported that residents were interested in engaging in activities related to their past hobbies and interests [21]. Therefore, these interests could be utilised to find income-generation activities.

This study highlights several important barriers and facilitators to focus on to improve the quality of care for older people in ACHs. Including two stakeholder groups (i.e., caregivers and older people) from a similar geographical setting is a strength of the study as it enables the pooling of ideas to understand the existing condition better. However, the study has limitations. In a multi-religious country, including only one religious group is considered a limitation as views of other religions would have expressed different perceptions. We relied on data given by caregivers when selecting study participants, which could lead to bias. Additionally, qualitative interpretations are subject to interpreter bias. Moreover, conducting a study in one province in Sri Lanka is a limitation. The context of health services and people's attitudes will differ from one province to another. Therefore, further research in different settings and provinces is needed.

This study identified barriers and facilitators for providing quality care for residents in ACHs from the perspective of residents and caregivers. The study has education, policy, and research implications. (a) education implications: The findings indicate the need to implement educational programmes for caregivers in collaboration with educational institutions to enhance the quality of care. (b) policy implications: Institutional authorities ought to provide capacity-building programmes for older people and caregivers introducing income generation activities to solve financial constraints. (c) research implications: Scholars need to do further studies in different settings to explore and enhance the quality of care. The findings also deserve greater attention by healthcare policymakers and social welfare authorities to take necessary measures to enhance the quality of life of older individuals in ACHs.

## Conclusion

Both residents and caregivers reported insufficient financial support on infrastructure, supplying medication and employing human resources as barriers to implementing quality care. Additionally, lack of referring system facilities, caregivers’ and residents’ insufficient knowledge of gerontological care and geriatric syndromes, lack of interest in being a caregiver at the ACHs, and lack of in-service programmes for caregivers were identified as barriers for quality care. Meanwhile, donations from philanthropists, supportive leadership, welfare benefits from the government authorities, and positive institutional values were the reported facilitators for quality care in ACHs. The available human and physical resources are insufficient to address the demanding needs of residents in ACHs, apart from the received donations and welfare benefits.

## Data Availability

The data used to support the findings of this study are available from the corresponding author upon request.
